# Efficacy of secukinumab in Takayasu arteritis with myocardial infarction complicated with generalized pustular psoriasis: A case report

**DOI:** 10.1097/MD.0000000000040994

**Published:** 2024-12-20

**Authors:** Tomoyuki Asano, Shuhei Yoshida, Naoki Matsuoka, Masato Ishikawa, Akihiko Sato, Shotaro Ogawa, Kenji Saito, Yuya Sumichika, Haruki Matsumoto, Jumpei Temmoku, Yuya Fujita, Shuzo Sato, Yasuchika Takeishi, Toshiyuki Yamamoto, Kiyoshi Migita

**Affiliations:** a Department of Rheumatology, Fukushima Medical University School of Medicine, Fukushima, Japan; b Department of Dermatology, Fukushima Medical University School of Medicine, Fukushima, Japan; c Department of Cardiovascular Medicine, Fukushima Medical University School of Medicine, Fukushima, Japan; d Department of Rheumatology, St. Francis Hospital, Nagasaki, Japan.

**Keywords:** generalized pustular psoriasis, interleukin 17, myocardial infarction, secukinumab, Takayasu arteritis

## Abstract

**Rationale:**

Takayasu arteritis (TAK) is an autoimmune disease that causes chronic inflammation targeting the aortic wall. Since many patients are resistant to steroid treatment, multiple immunosuppressants or interleukin-6 (IL-6) suppression therapy have served as treatment alternatives. However, there are very few reports on the effectiveness of biologics against inflammatory cytokines upstream of IL-6.

**Patient concerns:**

We present a case of TAK in a 51-year-old female presenting with a myocardial infarction. She had persistent carotid wall thickening despite glucocorticoid (GC) therapy, and IL-6 suppression therapy was being proactively considered. While the GC was being tapered, widespread pustules appeared all over her body.

**Diagnosis:**

TAK complicated with generalized pustular psoriasis (GPP).

**Interventions:**

Aside from GC, the patient was treated with secukinumab (SEC), an anti-IL-17A monoclonal antibody.

**Outcomes:**

Immediately after treatment with SEC, the pustules disappeared, and the thickening of the common carotid artery wall improved on ultrasound.

**Lessons:**

Since IL-17 is an important cytokine in the pathogenesis of TAK, anti-cytokine therapy targeting IL-17 may be effective for TAK.

## 1. Introduction

Takayasu arteritis (TAK) is a type of large vessel vasculitis that often affects the aorta and its trunk as well as the coronary and pulmonary arteries. The extravascular manifestations of TAK are complicated by gastrointestinal and cutaneous lesions, which may pose clinical problems. The cutaneous complications of TAK, such as erythema nodosum or pyoderma gangrenosum, are often treated with glucocorticoids (GCs), immunosuppressants, and biologics according to the therapeutic guidelines for TAK.^[1]^ Specifically, these immunosuppressants and biologics include methotrexate, azathioprine, tumor necrotizing factor (TNF) inhibitors, or interleukin-6 (IL-6) receptor inhibitors. These agents are selectively administered for refractory TAK, depending on the patient’s condition. In 1 report, tocilizumab (TCZ), an anti-IL-6 receptor inhibitor, helped prevent TAK recurrence and reduce GC use.^[2]^ However, in another study, immunosuppressive therapy with TCZ did not reach the primary endpoint of treatment for many patients,^[3]^ and the fundamental treatment of TAK remained unaddressed. A recent genome-wide analysis (GWAS) identified *IL-12B* as the gene susceptible to TAK.^[4]^ Molecular therapy targeting IL-12 molecules and IL-12-associated receptors has also been highly focused on.^[5]^ Meanwhile, IL-17 is an inflammatory cytokine produced downstream of IL-12 due to inflammation and is also known to be elevated in TAK.^[6]^ We report a unique case in which secukinumab (SEC), an anti-IL-17 monoclonal antibody, was influential in the treatment of TAK complicated by generalized pustular psoriasis (GPP).

## 2. Case report

In December 2021, a 51-year-old Japanese female was brought to our hospital for acute chest pain with abnormal vital signs. Six months prior, she was diagnosed with erythema nodosum at another hospital, which improved after oral prednisolone (PSL) at a dose of 20 mg/d, and so PSL was tapered and maintained at a dose of 5 mg/d. She had no notable medical or family history, including coronary disease risk factors. She had a height of 159 cm and weight of 42 kg. The patient was hypotensive, with a blood pressure of 62/40 mm Hg, whereas the rest of the vital signs were within normal limits (body temperature: 36.2°C, pulse rate: 62 beats/minute, and oxygen saturation: 100% on room air). Notably, there were 5 mm brown spots with subcutaneous indurations on both lower extremities, but the eruption activity was not intense because of the daily PSL (5 mg/d). Electrocardiogram revealed ST-elevation in leads I, aVL, aVR, and V1 to 6, while echocardiography revealed ischemic functional mitral regurgitation with severe left ventricular hypokinesis in the perfusion area of the left coronary artery. The patient was diagnosed with Killip class IV ST-elevation myocardial infarction and referred for emergency catheterization. Coronary angiography revealed subtotal occlusion of the ostial left main trunk, and emergent percutaneous coronary intervention (PCI) was performed. Supported by a left ventricular assist device, IMPELLA CP® (ABIOMED, Inc., Danvers, MA), a drug-eluting-stent, XIENCE Skypoint™ 4.0/8 mm (Abbott Vascular Japan Co., Ltd. Tokyo, Japan), was implanted into the ostial left main trunk, which resulted in no residual stenosis and achieved Thrombolysis in Myocardial Infarction Trial (TIMI) grade 3 coronary flow. Although the patient had a severe myocardial infarction with cardiogenic shock, she had a good clinical course after emergent PCI without worsening heart failure or progression of left ventricular remodeling, which could be attributed to the prompt introduction of IMPELLA CP® and re-vascularization via PCI.

Myocardial infarction due to a rheumatic disease, such as vasculitis, was suspected because the patient was a relatively young female without coronary risk factors. Her laboratory results are as follows, with normal values presented in parentheses: white blood cell count 8,400/µL (2,800–8,800), neutrophils 78% (44–74), hemoglobin 11.4 g/dL (11.6–14.0), platelet count 30.4 × 10^4^/µL (14.7–34.1 × 10^4^), brain natriuretic peptide 490.3 pg/mL (<18.4), aspartate transaminase 12 U/L (13–33), alanine transaminase 9 U/L (6–27), lactate dehydrogenase 221 U/L (119–229), alkaline phosphatase 72 U/L (115–359), blood urea nitrogen 17 mg/dL (8–22), creatinine 0.93 mg/dL (0.4–0.7), C-reactive protein (CRP) 1.19 mg/dL (<0.30), erythrocyte sedimentation rate 53 mm (3–15), interleukin (IL)-6 24.3 pg/mL (<7.0), serum amyloid-A 31.2 µg/mL (<8.0), matrix metalloproteinase-3 104 ng/mL (17.3–59.7), antinuclear antibodies < 1:160, rheumatoid factor < 5 IU/mL (<15), immunoglobulin (Ig) G 1,063 mg/dL (870–1,700), antiDNA antibodies 1.0 mg/dL (<10.0), myeloperoxidase antineutrophil antibodies < 0.2 mg/dL (<3.5), and proteinase-3 antineutrophil cytoplasmic antibodies < 0.6 mg/mL (<2.0). During treatment for heart failure, bilateral carotid artery ultrasound sonography (US) revealed circumferential wall thickening of the intima-media thickness (IMT; max IMT 1.6 mm) and elevated peak systolic velocity (PSV; PSV 131.7 cm/s), suggesting intrawall inflammation and stenosis of the carotid artery. Positron emission tomography with ^18^F-fluorodeoxyglucose computed tomography (^18^FDG-PET CT) showed no significant uptake in the large vessel wall. Although the patient did not meet the 1990 ACR classification criteria for TAK,^[7]^ the 2017 Isobe et al classification criteria for TAK was fulfilled.^[8]^ Finally, TAK was diagnosed, and oral PSL was administered at 30 mg daily. Since the TAK-induced vascular lesions were asymptomatic after PCI, PSL was gradually tapered while monitoring CRP to indicate treatment efficacy. Six weeks after PCI and PSL therapy, the patient was asymptomatic, with normal serum CRP levels. However, the US of the carotid artery showed no improvement with a max IMT of 1.5 mm, PSV of 161.3 cm/s, and resistance index (RI) of 1.6. Therefore, the patient was considered for additional immunotherapeutic treatment, such as TCZ for refractory TAK.

**Figure 1.**
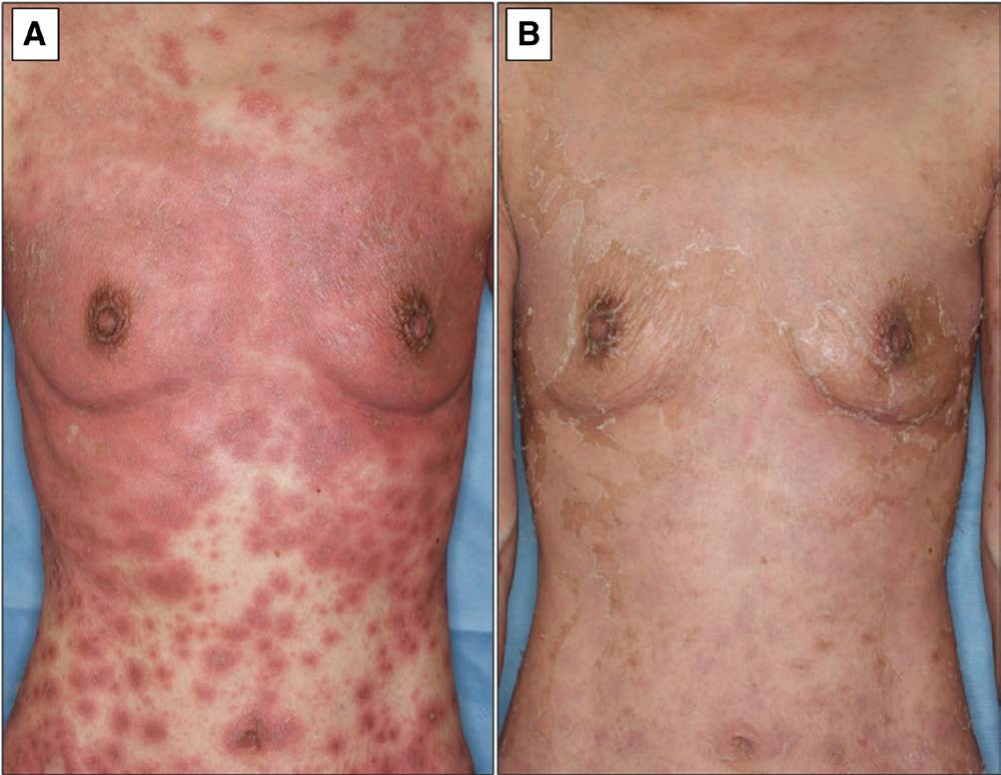
Skin appearance before and after secukinumab therapy. (A) Before secukinumab. Edematous and erythematous lesions on the trunk with small pustules, tending to coalesce partially. (B) After secukinumab. Remarkable disappearance of erythema with only slight peeling of the epidermis.

**Figure 2.**
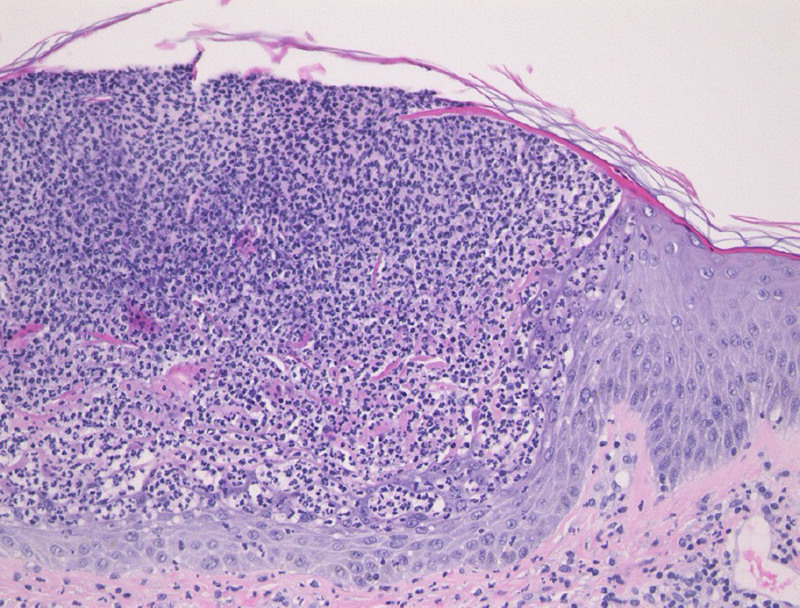
Histopathologic analysis of pustular lesions. Pustule formation was observed in the epidermis, and numerous neutrophil infiltrations were observed.

**Figure 3.**
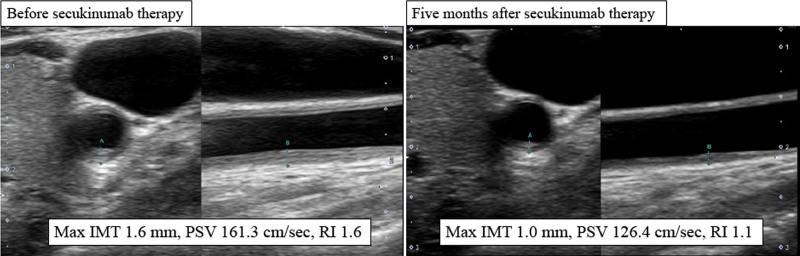
Changes in carotid artery ultrasound findings before and 5 months after secukinumab therapy. Five months after secukinumab treatment, ultrasound revealed improvement in the left common carotid artery wall thickening, as well as in blood flow velocity and vascular resistance. Left, transverse scan; right, longitudinal scan. IMT = intima-media thickness, PSV = peak systolic velocity, RI = resistance index.

Three months after admission, in March 2022, the patient developed a diffuse pustular rash on her extremities and trunk that spread rapidly. Diffuse edematous and erythematous lesions were seen on the trunk and extremities, with superficial small pustules on the margins, some of which had coalesced (Fig. 1A). This was accompanied by a modest increase in CRP (2.31 mg/dL). Subsequent skin biopsy revealed that pustule formation consisting of neutrophils in the epidermis, a pustular margin with Kogoj spongiform abscess, and infiltration of lymphocytes and neutrophils around the blood vessels in the superficial dermis (Fig. 2). Thus, the patient was additionally diagnosed with GPP, which was treated with the anti-IL-17A antibody agent SEC, starting with a loading dose of 5 consecutive weekly injections, followed by monthly self-injection (300 mg/mo). Four days after SEC administration, there was marked improvement of the diffuse erythema of the extremities and trunk (Fig. 1B). Moreover, the carotid US findings 6 weeks after SEC showed marked improvement in the IMT and PSV of the common carotid artery, indicating that anti-IL-17A antibody therapy for GPP also suppressed the disease activity of TAK (Fig. 3). After 53 months of SEC therapy, the PSL dose was tapered to 8 mg daily, and the GPP and TAK remained in clinical remission.

## 3. Discussion

We present a case of TAK that first presented as a myocardial infarction and subsequently developed GPP as a complication. Cutaneous and gastrointestinal lesions are the frequent comorbid extravascular lesions associated with TAK. In particular, skin lesions occur in 18% to 31.6% of cases, with the most common being erythema nodosum.^[9,10]^ Erythema nodosum encompasses a relatively broad spectrum of diseases; its phenotypes can be caused by an allergic reaction to infections, drugs, and even systemic diseases such as TAK.^[11]^ Psoriasis vulgaris complicated with TAK is relatively common,^[9]^ but to our knowledge, this is the first report of GPP associated with TAK which was treated with SEC.

In terms of epidemiology, TAK is common in the young Asian population, and aortic regurgitation is the most cardiological complication with aortic lesions.^[12]^ Coronary artery lesions are also frequently observed in 7% to 10% of TAK patients.^[13]^ We previously reported the case of an 18-year-old female with TAK complicated by angina due to coronary artery lesions.^[14]^ For severe stenosis of the left and right main coronary arteries, she was given immunosuppressive multitherapy (PSL, methotrexate, and TCZ) and optimal medical treatment, including a beta-blocker, antiplatelet, and statin. However, the severe stenosis of the left and right main coronary arteries did not allow for sufficient blood flow.^[14]^ Despite adequate immunosuppressive therapy, there are cases where TAK coronary artery lesions are exacerbated or do not improve due to age, a delay in diagnosis and treatment, TAK activity, arteriosclerotic disease comorbidity, and smoking.^[15]^

One of the critical pathological roles of TAK is the production of inflammatory cytokines such as IL-6 and interferon (IFN)-γ from CD4^+^ T cells in the adventitia of the vascular wall. These inflammatory cytokines induce activated macrophages and the differentiation of giant cells.^[16]^ Terao et al reported a strong correlation between the *IL-12B* region on chromosome 5 and the human leukocyte antigen (*HLA)-B* region on chromosome 17 (proxies for HLA-B*52:01) in TAK patients, suggesting their synergistic involvement in the development of TAK.^[4]^ Additionally, in patients with *IL-12B* risk alleles, serum IL-12p70 concentrations and cell supernatant IL-12/23p40 and IL-12p70 concentrations were high, suggesting that *IL-12B* polymorphisms are associated with the onset of Takayasu arteritis through increased IL-12/23p40 protein concentrations.^[17]^ Based on these findings, Terao et al conducted a pilot clinical trial of ustekinumab (UST), a recombinant IgG1 monoclonal antibody agent against the p40 subunit of human IL-12/23, in 3 patients with highly active TAK, and they reported the safety and efficacy of the treatment.^[18]^ Unfortunately, UST is not routinely given to TAK patients because it is not covered by health insurance in Japan. However, Suga et al administered UST in a patient with Crohn's disease and TAK and achieved a good response (note: UST is covered by health insurance for Crohn's disease in Japan).^[19]^

IL-17, which is produced by Th17 cells, recruits monocytes or neutrophils to local tissues.^[20]^ The *IL-12B* gene encodes p40, the common subunit of IL-12 and IL-23, and IL-23 induces differentiation of naive T cells into Th17 cells.^[21]^ The released IL-17 aids in neutrophil migration into the vascular wall in patients with atherosclerosis.^[22]^ Moreover, IL-17 activation leads to the activation of many downstream cytokines/chemokines, such as IL-1β, IL-6, IL-8, IL-21, TNF-β, and monocyte chemotactic protein-1 (MCP-1).^[23]^ The IL-17-mediated inflammatory microenvironment strongly promotes fibrosis in the adventitia of arteries in patients with TAK.^[24]^ Therefore, SEC, an anti-IL-17A antibody, has strong potential as a novel therapeutic agent for IL-17-dependent TAK, and more studies are expected on this drug in the future.

In conclusion, we report a case where SEC, an anti-IL-17A antibody agent, demonstrated long-term efficacy in suppressing both TAK complicated by GPP. IL-17 is implicated in the pathogenesis of TAK, and anti-cytokine therapy targeting IL-17 has been discussed as a potential treatment in various journals. However, this is the first report to demonstrate the effectiveness of SEC, specifically in TAK. Many patients with refractory TAK require long-term GC therapy to control disease activity, often suffering from GC-related side effects. Anti-cytokine therapy options for TAK should expand in the future, potentially offering alternative treatments for patients struggling with the adverse effects of prolonged GC use.

## Acknowledgments

We thank Enago (http://enago.jp) for the English language review. We also sincerely thank Ms. Mitsuko Matsuda for her assistance in evaluating the carotid artery by ultrasound.

## Author contributions

**Conceptualization:** Tomoyuki Asano.

**Data curation:** Tomoyuki Asano, Shuhei Yoshida, Naoki Matsuoka.

**Formal analysis:** Tomoyuki Asano.

**Investigation:** Tomoyuki Asano.

**Methodology:** Tomoyuki Asano.

**Project administration:** Tomoyuki Asano.

**Resources:** Tomoyuki Asano.

**Supervision:** Kiyoshi Migita.

**Validation:** Tomoyuki Asano, Masato Ishikawa, Akihiko Sato.

**Visualization:** Tomoyuki Asano.

**Writing – original draft:** Tomoyuki Asano.

**Writing – review & editing:** Tomoyuki Asano, Masato Ishikawa, Akihiko Sato, Shotaro Ogawa, Kenji Saito, Yuya Sumichika, Haruki Matsumoto, Jumpei Temmoku, Yuya Fujita, Shuzo Sato, Yasuchika Takeishi, Toshiyuki Yamamoto, Kiyoshi Migita.
